# *In vivo* accurate detection of the liver tumor with pharmacokinetic parametric images from dynamic fluorescence molecular tomography

**DOI:** 10.1117/1.JBO.27.7.070501

**Published:** 2022-07-09

**Authors:** Fei Liu, Peng Zhang, Zeyu Liu, Fan Song, Chenbin Ma, Yangyang Sun, Youdan Feng, Yufang He, Guanglei Zhang

**Affiliations:** a Beijing Information Science & Technology University, Advanced Information and Industrial Technology Research Institute, Beijing, China; b Beihang University, Beijing Advanced Innovation Center for Biomedical Engineering, School of Biological Science and Medical Engineering, Beijing, China

**Keywords:** dynamic fluorescence tomography, pharmacokinetic imaging, liver tumor detection

## Abstract

**Significance:**

Pharmacokinetic parametric images in dynamic fluorescence molecular tomography (FMT) can describe three-dimensional (3D) physiological and pathological information inside biological tissues, potentially providing quantitative assessment tools for biological research and drug development.

**Aim:**

*In vivo* imaging of the liver tumor with pharmacokinetic parametric images from dynamic FMT based on the differences in metabolic properties of indocyanine green (ICG) between normal liver cells and tumor liver cells inside biological tissues.

**Approach:**

First, an orthotopic liver tumor mouse model was constructed. Then, with the help of the FMT/computer tomography (CT) dual-modality imaging system and the direct reconstruction algorithm, 3D imaging of liver metabolic parameters in nude mice was achieved to distinguish liver tumors from normal tissues. Finally, pharmacokinetic parametric imaging results were validated against *in vitro* anatomical results.

**Results:**

This letter demonstrates the ability of dynamic FMT to monitor the pharmacokinetic delivery of the fluorescent dye ICG *in vivo*, thus, enabling the distinction between normal and tumor tissues based on the pharmacokinetic parametric images derived from dynamic FMT.

**Conclusions:**

Compared with CT structural imaging technology, dynamic FMT combined with compartmental modeling as an analytical method can obtain quantitative images of pharmacokinetic parameters, thus providing a more powerful research tool for organ function assessment, disease diagnosis and new drug development.

## 1 Introduction

Dynamic fluorescence molecular tomography (FMT) is a powerful imaging tool that can resolve the three-dimensional (3D) distribution of fluorophores in small animals and has been extensively used for imaging studies of pharmacokinetics parameters.^1^ Compartment modeling is a classic approach for pharmacokinetic analysis, and the exchange rates of materials between different compartments are defined as pharmacokinetic parameters.^2^ The combination of dynamic FMT and compartmental modeling can provide 3D images of pharmacokinetic parameters *in vivo* for tumor detection, drug development, and metabolism studies.^3^ However, dynamic FMT remains challenging in imaging pharmacokinetic parameters of small animal organs due to the severe ill-posed nature of the inverse problem, making it challenging to determine the location of specific organs and perform the pharmacokinetics analysis.^4^ Furthermore, when different tissues or organs are assigned with the same average optical parameters, inaccurate FMT forward models can also be generated, further reducing the accuracy of the pharmacokinetic parameter analysis.

Previous studies have shown that the prior information of anatomical structures provided by structural imaging techniques such as computer tomography (CT) or magnetic resonance imaging (MRI) can be used to construct more accurate forward physical models by assigning heterogeneous optical parameters to different tissues or organs after segmenting the structural information; Moreover, the structural information can be used to construct *a priori* models, which can guide and constrain the solution process of the inverse problem and improve the reconstruction quality.^5^^–^^7^ Therefore, a hybrid dual-modality FMT/CT system has been employed in this work to image the pharmacokinetic parameters.

The liver is an essential organ for studying metabolism parameters in dynamic FMT.^8^ Indocyanine green (ICG) is a clinically approved reagent for liver function assessment by the Food and Drug Administration. Meanwhile, ICG is a widely used optical fluorophore in the near-infrared range for FMT. Therefore, liver tumors can be distinguished *in vivo* according to the different metabolic properties of ICG in normal and liver cancer cells by pharmacokinetic parameters imaging with the help of dynamic FMT.

In this letter, dynamic FMT is conducted to detect liver tumors *in vivo*. First, the orthotopic liver tumor mouse model was constructed. Then, 3D imaging of ICG metabolic parameters of the mouse liver was achieved with the help of the FMT/CT dual-modality imaging system and a direct reconstruction algorithm, and liver tumors can be discriminated based on the reconstructed parametric images according to the different metabolic properties of ICG in liver cancer cells. Finally, the parametric imaging results were validated against *in vitro* anatomical results.

## 2 Materials and Methods

### 2.1 FMT/CT Dual-Modality Imaging System

**Fig. 1:**
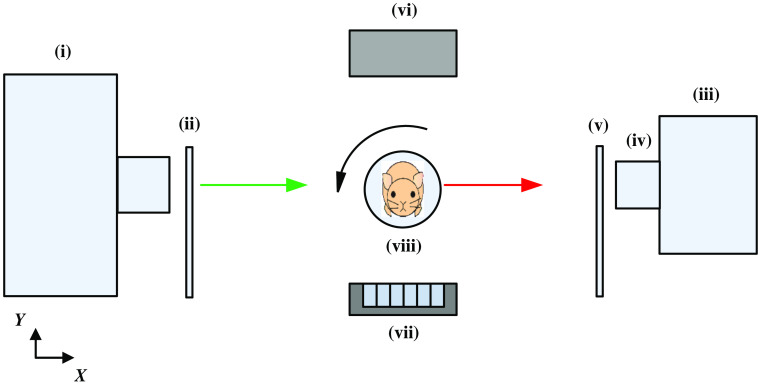
Schematic diagram of the FMT/CT dual-modality imaging system.

The dual-modality system consists of two imaging modalities, FMT and CT, which are placed vertically at a 90-deg angle.^9^ The schematic diagram of the system is shown in Fig. 1. A laser (i) (Asahi Spectra, Torrance, California) is employed to excite the imaged small animal (viii). Full-angle measurements are implemented by a rotation stage working under a step-by-step mode. Data acquisition is performed by an electron-multiplying charge-coupled device camera (iii) (iXon DU-897, Andor Technologies, Belfast, Northern Ireland) coupled with a 35-mm f/1.6 lens (iv) (C3514-M, Pentax, Japan). Corresponding excitation filter (ii) and emission filter (v) pairs are employed for data acquisition. The x-ray source (vi) is an XTF5011 X-ray tube (Oxford Instrument, California), and the x-ray detector (vii) is a 1056×1056  pixel CMOS array detector (Hamamatsu, Shizuoka, Japan) for acquiring CT projection data.

In the dual-modality system, the laser was used as the excitation light source, and the shape of the excitation light source was a linear excitation light with a length of 2 cm and a width of 2 mm. The field of view of the charged-coupled device (CCD) camera was 8.2  cm×8.2  cm. The CCD pixels used for fluorescence projection data acquisition were 2×2, the exposure time was set as 0.5 s, and the excitation light power was 1.5 mW.

### 2.2 Experiments

#### 2.2.1 Cell culture

HepG2 cells of a human hepatocellular carcinoma cell line were cultured in Dulbecco’s modiﬁed Eagle’s Medium (Invitrogen, Carlsbad, California) with 10% fetal bovine serum (Gibco) in a humidified incubator at 37°C in a 5% atmosphere. Cells were harvested by trypsinization and resuspended in phosphate buffer solution (PBS) before injection.

#### 2.2.2 Animal studies

Animal experiments were conducted under the protocol approved by the Institutional Animal Care and Use Committee of Beihang University. An 8-week BALB/c mice (female) mouse was anesthetized by intraperitoneal injection of 2% sodium pentobarbital solution at a dose of 0.3 ml/100 g body weight. The mouse was placed in a supine position and fixed on a mouse plate. The abdominal skin was wiped with iodine and alcohol for routine disinfection. An incision was made with surgical scissors along the left upper abdominal pararectal line and peritoneum to expose the liver, and 0.02 ml of $4\times 10^{5}$ HepG2 cells in PBS was injected into the right lobe of the liver. After inoculation, the abdominal incision was closed with surgical sutures, and gentamycin eye ointment was applied to the suture to prevent infection of the incision.

#### 2.2.3 Data acquisition

The data acquisition process for FMT/CT dual-modality imaging system is as follows. During the acquisition of fluorescence projection data for dynamic fluorescence molecular imaging, the imaged mouse was rotated continuously for multiple frames on the rotating platform, and a total of 110 frames were collected in this study. The fluorescence data from each frame was used for FMT reconstruction and to obtain the 3D image. After the fluorescence projection data acquisition was completed, the data acquisition of the CT system was performed to obtain the anatomical structure of the imaged mouse. For the acquisition of CT projection data, only one rotation of the imaged mouse was required.

Additionally, to achieve accurate registration of the imaging results of the FMT and CT systems, the metal marker dot was attached to the body surface of the nude mouse, and a white light image and a CT projection image were acquired separately to provide the height information required for the registration of the two imaging systems.

### 2.3 Pharmacokinetic Parameter Reconstruction Method

The compartment modeling can be expressed by the differential equation as follows:

$$\left\{ \begin{array}{l} \frac{dc_{1}(t)}{dt}=f_{10}+\overset{n}{\underset{i=2}{\sum }}[k_{1i}c_{i}(t)-k_{i1}c_{i}(t)]-k_{01}c_{1}(t)\\ \frac{dc_{i}(t)}{dt}=k_{i1}c_{i}(t)-k_{1i}c_{i}(t) \end{array},\right.$$ (1)

where $c_{1}$ denotes the concentration of the fluorescent probe in the blood, $c_{i}$ (i=2∼n) denotes the concentration of the fluorescent probe in different tissues and organs, $k_{i1}$ denotes the rate coefficient of fluorescent probe inflow from blood to organ i (i=2∼n), $k_{1i}$ denotes the rate coefficient of fluorescent probe inflow into the blood from different organs i (i=2∼n), $k_{01}$ denotes the rate coefficient of the fluorescent probe excreted from the blood, and $f_{10}$ is the rate at which the fluorescent probe is injected into the blood.

By solving Eq. (1), the metabolism curves of ICG in different tissues and organs can be obtained:

$$C_{i}(t)=-A_{i}e^{-\alpha_{i}t}+B_{i}e^{-\beta_{i}t},$$ (2)

where $C_{i}(t)$ is the ICG concentration at the time of t; A, B, α, and β are the metabolic parameters that can characterize the pharmacokinetic properties of each tissue and organ. α denotes the uptake rate of the fluorescent probe by the tissue, β denotes the excretion rate of the fluorescent probe by the tissue, A and B denote the concentration gain of the model, which is related to the concentration of the fluorescent probe.

To obtain the metabolic parameters of dynamic FMT reconstruction, the internal anatomy of nude mice was first obtained by CT as *a priori* information for the dynamic FMT reconstruction algorithm. Then, the dynamic FMT imaging region of the mouse was divided into four parts: lung, liver, kidney, and other tissues based on the structural prior information provided by the CT results, and finally, the corresponding optical parameters were assigned to the different organs of the mice based on Table 1 to construct the heterogeneous forward model.

**Table 1:** Heterogeneous optical parameters in mice.^10^

Organs	$\mu_{a}$ (${\mathrm{cm}}^{-1}$)	$\mu_{s}^{\prime }$ (${\mathrm{cm}}^{-1}$)
Lung	0.25	30
Liver	0.5	13
Kidney	0.175	20.9
Other tissues	0.3	10

Then, the double exponential curve model obtained from Eq. (2) is substituted into the heterogeneous forward model, and the results of the forward prediction function are obtained as follows:

$$f(X)=\overset{N}{\underset{j=1}{\sum }}Wx(t_{k})=\overset{N}{\underset{j=1}{\sum }}W[-A\;\exp(-\alpha t_{k})+B\;\exp(-\beta t_{k})].$$ (3)

Finally, the inverse problem of Eq. (3) is solved by the full-direct FMT method^9^ to reconstruct the parametric images. This method can make full use of the temporal correlations of boundary measurements to model the high temporal variation of fluorophore concentration. Exactly, the objective function of the direct reconstruction algorithm can be obtained by substituting Eq. (3) into the Laplace regularization matrix:

$$\Psi (X)={\left\|y-f(X)\right\|}_{2}^{2}+\lambda_{1}{\left\|LA\right\|}_{2}^{2}+\lambda_{2}{\left\|LB\right\|}_{2}^{2}+\lambda_{3}{\left\|L\alpha \right\|}_{2}^{2}+\lambda_{4}{\left\|L\beta \right\|}_{2}^{2},\;={\left\|y-f(X)\right\|}_{2}^{2}+\overset{4}{\underset{i=1}{\sum }}\lambda_{i}{\left\|Lx_{i}\right\|}_{2}^{2},$$ (4)

where X denotes the fluorophore concentration distribution, and L denotes the Laplace matrix, and A, B, α, and β are the pharmacokinetic parametric images to be reconstructed. The pharmacokinetic parameters A and B have an arbitrary unit (a.u.), while α ($\min^{-1}$) and β ($\min^{-1}$) are the uptake and excretion rates of fluorophores, which have physiological significance for quantitative evaluation of organ function.^11^ In this letter, the regularization parameters were set as $\left[\lambda_{1},\lambda_{2},\lambda_{3},\lambda_{4}]=[2\times 10^{6},5\times 10^{6},5\times 10^{3},1\times 10^{4}\right]$, and the number of iterations was 200. The CT data were reconstructed using the Feldkamp–Davis–Kress algorithm,^12^ and the size of the reconstructed voxels was $0.1\times 0.1\times 0.1\;{\mathrm{mm}}^{3}$.

## 3 Results and Discussion

**Fig. 2:**
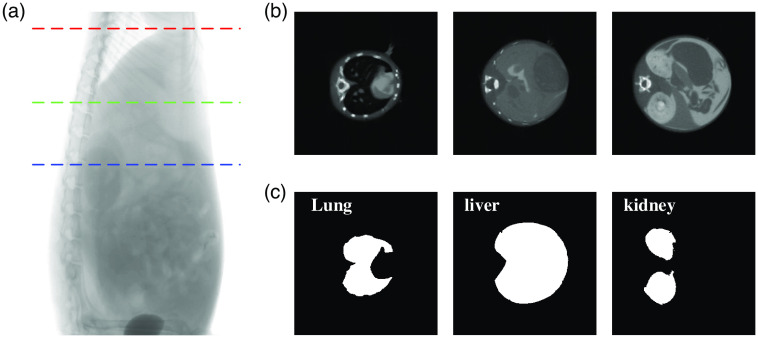
CT imaging results of the tumor-bearing nude mouse. (a) A representative CT projection image of the tumor-bearing mouse. (b) Three cross-sectional images of the CT results. (c) CT segmentation results of lung, liver, kidney, and other tissues.

The anatomical imaging results of the mouse are shown in Fig. 2. Figure 2(a) shows a representative CT projection image of the tumor-bearing mouse. Figure 2(b) shows three crosssectional images of the CT reconstruction results of the tumor-bearing mouse with the heights corresponding to the positions of the red, green, and blue dashed lines in Fig. 2(a), respectively. The whole imaging area of the mouse was manually segmented into lung, liver, kidney, and other tissues [Fig. 2(c)] according to the gray values of the CT images, and these segmentation results will be used as structural *a priori* information for the metabolic parameters of the FMT reconstruction.

**Fig. 3:**
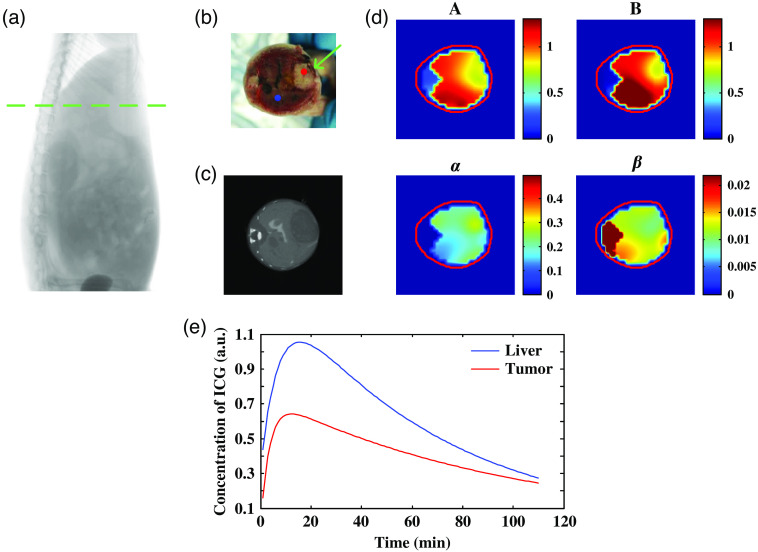
Imaging results of metabolic parameters in liver tumors. (a) CT projection image. (b) Cryosection image of mouse with a green arrow indicating the tumor location. (c) Cross-sectional view of CT imaging results. (d) The metabolic parameter images A (a.u.), B (a.u.), α ($\min^{-1}$), and β ($\min^{-1}$) of the mouse. (b)–(d) correspond to the heights represented by the green-dashed lines in (a). (e) ICG metabolic curves of the liver.

Figure 3 shows the dynamic FMT reconstruction results of liver metabolic parameters. Figure 3(a) shows a CT projection, and the green dotted lines indicate the corresponding heights of the reconstructed images in Figs. 3(b)–3(d). Figure 3(b) shows an image of a frozen mouse section obtained after imaging, the height of which was approximately matched to the height of the tomographic results by manual marking. The actual location of the tumor in the liver is shown by the green arrow in Fig. 3(b). The liver tumor region can be clearly identified from the imaging results of metabolic parameters shown in Fig. 3(d). Moreover, based on the reconstructed metabolic parameter images, four metabolic parameter images A, B, α, and β can be obtained. By substituting the parameter values into Eq. (2), we can quantitatively recover the ICG metabolic curve of the liver. As shown in Fig. 3(e), the blue curve represents the ICG metabolic curve at the location of the blue point in the normal liver region in Fig. 3(b), and the red curve represents the ICG metabolic curve at the location of the red point in the tumor region in Fig. 3(b). It can be found that compared with the normal liver tissue, the uptake rate of ICG is increased and the excretion rate of ICG is decreased in the tumor area, which is consistent with the theoretical expectation.

## 4 Conclusion

Pharmacokinetic parameters can describe the dynamics of drug absorption, metabolism and excretion processes and enable quantitative monitoring of physiological properties such as permeability and perfusion rates of tissues and organs. Metabolic parameter imaging technology can visualize the pharmacokinetic parameters of each voxel in the imaging area, allowing a more intuitive description of the physiological processes of drug absorption and excretion in different tissues and organs, which is of great significance for physiological and pathological studies.

In this letter, the pharmacokinetic parameters of ICG have been monitored through a hybrid FMT/CT system and the direct reconstruction algorithm. Although liver tumors can also be directly observed from CT imaging results, as a structural imaging technique, the functional state of their tissues and organs cannot be reflected in the CT images. As a functional imaging technique, quantitative assessment of liver function can be obtained by pharmacokinetic parametric images derived from dynamic FMT. Furthermore, ICG metabolic curves at any location in the liver can be achieved according to the reconstructed pharmacokinetic parameters. Compared with CT structural imaging, the imaging of metabolic parameters with dynamic FMT can obtain quantitative metabolic parameter images, providing a more powerful research tool for organ function assessment, disease diagnosis, and new drug development. Future work will focus on imaging pharmacokinetic parameters of pathological livers and other organs.
